# Growth Differentiation Factor 6 As a Putative Risk Factor in Neuromuscular Degeneration

**DOI:** 10.1371/journal.pone.0089183

**Published:** 2014-02-28

**Authors:** Michèle G. DuVal, Matthew J. H. Gilbert, D. Ezekiel Watson, Tanja C. Zerulla, Keith B. Tierney, W. Ted Allison

**Affiliations:** 1 Department of Biological Sciences, University of Alberta, Edmonton AB, Canada; 2 Centre for Prions and Protein Folding Disease, University of Alberta, Edmonton AB, Canada; 3 Department of Medical Genetics, University of Alberta, Edmonton AB, Canada; University of Edinburgh, United Kingdom

## Abstract

Mutation of *Glass bottom boat*, the *Drosophila* homologue of the bone morphogenetic protein or growth/differentiation factor (BMP/GDF) family of genes in vertebrates, has been shown to disrupt development of neuromuscular junctions (NMJ). Here we tested whether this same conclusion can be broadened to vertebrate BMP/GDF genes. This analysis was also extended to consider whether such genes are required for NMJ maintenance in post-larval stages, as this would argue that BMP genes are viable candidates for analysis in progressive neuromuscular disease. Zebrafish mutants harboring homozygous null mutations in the BMP-family gene *gdf6a* were raised to adulthood and assessed for neuromuscular deficits. Fish lacking *gdf6a* exhibited decreased endurance (∼50%, p = 0.005) compared to wild type, and this deficit progressively worsened with age. These fish also presented with significantly disrupted NMJ morphology (p = 0.009), and a lower abundance of spinal motor neurons (∼50%, p<0.001) compared to wild type. Noting the similarity of these symptoms to those of Amyotrophic Lateral Sclerosis (ALS) model mice and fish, we asked if mutations in *gdf6a* would enhance the phenotypes observed in the latter, i.e. in zebrafish over-expressing mutant Superoxide Dismutase 1 (SOD1). Amongst younger adult fish only bigenic fish harboring both the SOD1 transgene and *gdf6a* mutations, but not siblings with other combinations of these gene modifications, displayed significantly reduced endurance (75%, p<0.05) and strength/power (75%, p<0.05), as well as disrupted NMJ morphology (p<0.001) compared to wild type siblings. Bigenic fish also had lower survival rates compared to other genotypes. Thus conclusions regarding a role for BMP ligands in effecting NMJ can be extended to vertebrates, supporting conservation of mechanisms relevant to neuromuscular degenerative diseases. These conclusions synergize with past findings to argue for further analysis of *GDF6* and other *BMP* genes as modifier loci, potentially affecting susceptibility to ALS and perhaps a broader suite of neurodegenerative diseases.

## Introduction

Development, growth and stabilization of neuromuscular junctions (NMJ) in larval *Drosophila* require the bone morphogenetic protein (BMP) gene *glass bottom boat (gbb)* [1]. Here we tested whether this same conclusion can be broadened to vertebrates, and extended this analysis to consider whether such genes are required for vertebrate NMJ maintenance in post-larval stages. *Gbb* is the *Drosophila* homolog of a family of vertebrate genes including the BMP and growth/differentiation factor ligands (BMP/GDF Family), which is itself a sub-family of the transforming growth factor β (TGFβ) genes [1]–[3]. Extending their role to encompass vertebrate NMJs would embolden speculation that mutations in BMP/GDF genes can sensitize patients to progressive late-onset neuromuscular disease. This affirmation would support the contended relevance of several intriguing cellular and molecular mechanisms of neuromuscular degeneration, derived from *Drosophila* studies (see Discussion), that are hypothesized to impinge upon the development of treatments or diagnostics of neuromuscular disease.

Indeed the synthesis of disparate literature by several authors has recently suggested that mutations in BMP/GDFs are good candidates for sensitizing patients to amyotrophic lateral sclerosis (ALS), if not representing causal instigators of disease etiology [4], [5]. This suggestion was based not only on the aforementioned role of *Gbb* [1], [2], but also upon the requirement for the proteins constituting *Gbb* receptors in NMJ development [6]–[8]. BMP/GDFs have also been implicated in ALS progression via a separate line of inquiry, in that a *Drosophila* model of familial ALS8 demonstrates disrupted BMP signaling at their NMJs; Thus mutations in *VAPB* (vesicle-associated membrane protein B) cause ALS8 [9], [10] and altering *VapB* disrupts BMP signaling at the *Drosophila* NMJ [11]. A role for BMP/GDFs in other neuromuscular diseases has also been proposed, including Spinal Muscular Atrophy, Hereditary Spastic Paraplegias, Multiple Sclerosis and Huntington's Disease [4].

BMPs are most widely recognized for their fundamental roles in development across vertebrates, including patterning the dorsoventral axis of the body, CNS and retina [12]–[17]. BMP/GDF proteins heterodimerize or homodimerize to signal through BMP receptors, and canonically through phosphorylating SMAD proteins, though several other signaling cascades can be important in many instances [18].

ALS is a progressive neuromuscular disease caused by motoneuron loss, though the etiology of motoneuron death is unknown. Candidate causes include glutamate excitotoxicity, oxidative stress, and RNA processing defects. Symptoms in patients, recapitulated in animal models overexpressing mutant SOD1, include progressive muscle weakness and decreased endurance, altered gait, motor neuron death and progressive paralysis. Genetics of familial ALS (fALS) include lesions in TARDBP, FUS/TLS, C9orf72 and SOD1. Identification of misfolded SOD1 in sporadic ALS argues for a central role for SOD1 in ALS regardless of genetic or environmental initiators [19]. SOD1^G93A^ mice are an indispensable staple in the ALS field because they exhibit many etiological similarities to, and present with disease progression that has fidelity to, clinical ALS presentation. Zebrafish, with conserved CNS and motoneuron physiology/genetics, have proven very useful to study the genetic relationships between fALS genes via loss-of-function analyses [20]–[27]. Furthermore, toxic gain-of-function zebrafish have been generated overexpressing mutant SOD1 and that recapitulate all hallmark attributes of fALS [28], [29]. A substantial percentage of fALS remains to be explained, and genes that modify such susceptibility have potential to be influential in late-onset disorders with complex interactions of genetics and/or environmental factors.

The proposed requirement for BMP/GDF6 family genes in vertebrate NMJs is supported by at least three previous lines of inquiry. First, disruption of intracellular trafficking led to NMJ deficits in zebrafish mutants of *atlastin*, and it was argued that this deficit was due to disrupted trafficking of BMP Receptors [30]. Second, BMPs and BMP Receptors are increased in expression at NMJs during recovery from experimentally induced traumatic injury in mice [5]. Third, a role for TGFβ/BMP/GDF signaling in ALS progression is suggested by the increase in pSMADs in ALS inclusions observed in both patient pathology and mouse models [31]–[33]. Although these lines of evidence are encouraging and suggestive, a role for BMP ligands in vertebrate neuromuscular degeneration remains unaddressed.

Our recent success in raising topical mutant zebrafish to adulthood [34] enabled us to address the questions above regarding a role for BMPs in the maintenance of vertebrate NMJs. Zebrafish harboring a null mutation in a homolog of *GDF6* were outcrossed, establishing a viable and fecund line of homozygous *gdf6a* mutants. Increased cell death in at least some CNS neurons was reported during early development of these mutants [34]. This encouraged us to explore a potential role for *gdf6a* in neuromuscular degeneration, which would potentially implicate BMP/GDF6 genes as candidate loci in neuromuscular disease. We document progressive neuromuscular deficits in these mutants, including reduced muscle endurance, neuromuscular junction anomalies and loss of spinal motor neurons. Further, this mutation in *gdf6a* increased disease severity in a zebrafish model of ALS that over-expresses mutant SOD1. These data integrate to compel *GDF6* as a candidate locus that increases susceptibility to or progression of ALS.

## Results

### Gdf6^−/−^ zebrafish do not display motoneuron axonopathy in early development

**Figure 1:**
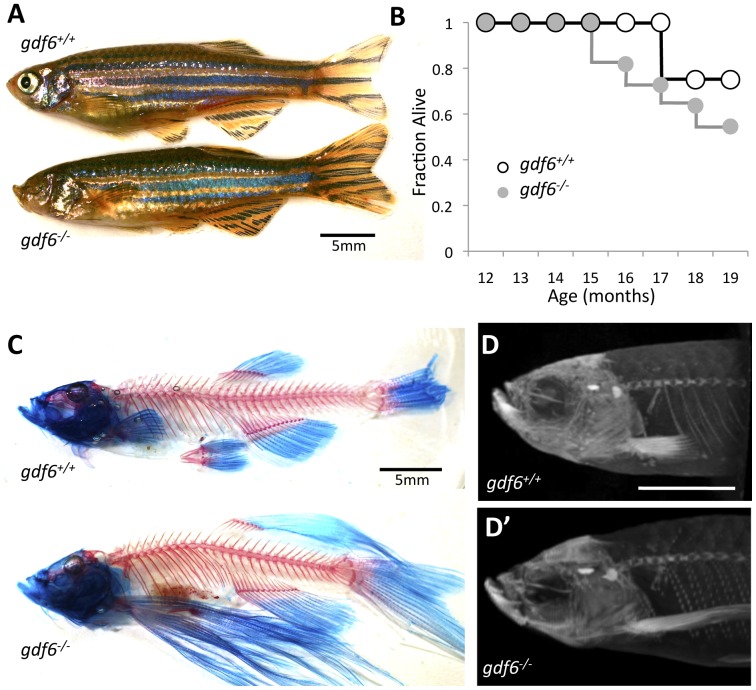
Zebrafish harboring homozygous mutations in *gdf6* can be raised to adulthood and do not display overt skeletal defects. **A.** *gdf6^−/−^* fish are viable into adulthood, exhibit variably penetrant microphthalmia and normal body morphology. **B.** *gdf6^−/−^* fish exhibit somewhat decreased survival compared to *gdf6^+/+^* siblings (n = 11 *gdf6^−/−^* fish; n = 4 *gdf6^+/+^* siblings). **C, D.** *gdf6^−/−^* fish lack overt skeletal phenotypes, as revealed by (C) clearing and staining or by (D) microCT analysis. The latter is further represented as Supplemental Movie S1. Scale bars are 5 mm. A variety of fin morphologies were present in the fish examined, but these were neither different between experimental groups (genotypes) nor a significant covariant with swim performance (see Results).

Because *Gdf6^−/−^* mice are not viable beyond embryonic stages [34], we considered alternate animal models and asked if zebrafish deficient in a *GDF6* homologue might present with neuromuscular deficits akin to ALS. Our recent work employed several generations of out-crosses to successfully isolate a viable line of homozygous null *gdf6a^s327/s327^* fish (ZFin ID: ZDB-ALT-050617-10) that can be raised to adulthood [34]. This nonsense mutation occurs in the pro-domain, at residue 55, abrogating production of mature Gdf6a ligand and herein we refer to these mutants as *gdf6a^−/−^*. These fish display microphthalmia [34], but are otherwise normal in development (Figure 1A), adult morphology, and fecundity. A decreased longevity in *gdf6a^−/−^* fish compared to their siblings was noted, not reaching statistical significance in a small cohort (Figure 1B).

To assess if *gdf6a^−/−^* embryos display motoneuron disease, we utilized sensitive assays of motoneuron disease/ALS that are deployed frequently in embryonic zebrafish, consisting of examining branching and pathfinding defects in GFP-positive primary motoneurons of transgenic fish [20]–[22], [24]–[27]. *gdf6a^−/−^* embryos did not display a significant increase in motoneuron pathfinding or axonopathies as determined in a transgenic background that enables sensitive detection of motoneuron morphology based on GFP fluorescence; both *gdf6a^−/−^* and siblings had a low rate of abnormalities in this scoring system (Supplemental Figure S1; p≥0.315, n≥9 larvae per genotype). We confirmed that this assay was sensitive to ALS-related genetic lesions in our hands by delivering mRNA encoding human SOD1 with or without mutations associated with familial ALS (5.7 and 0.18 primary motoneurons affected per fish injected with SOD1^A4V^ or SOD1^WT^, respectively p<0.05), consistent with past results [26], [27].

Considering ALS is a late-onset disease, it was of interest to characterize adult *gdf6a^−/−^* zebrafish with respect to ALS-like phenotypes. We examined fish at 9 to 18 months old (zebrafish are considered to be ‘adult’ at sexual maturity, ∼3 months old and can thrive for 3–5 years of age) regarding muscle endurance, gait, motoneuron abundance and character of their neuromuscular junctions.

### gdf6a loss in adult zebrafish leads to neuromuscular disease, including deficits in endurance

**Figure 2:**
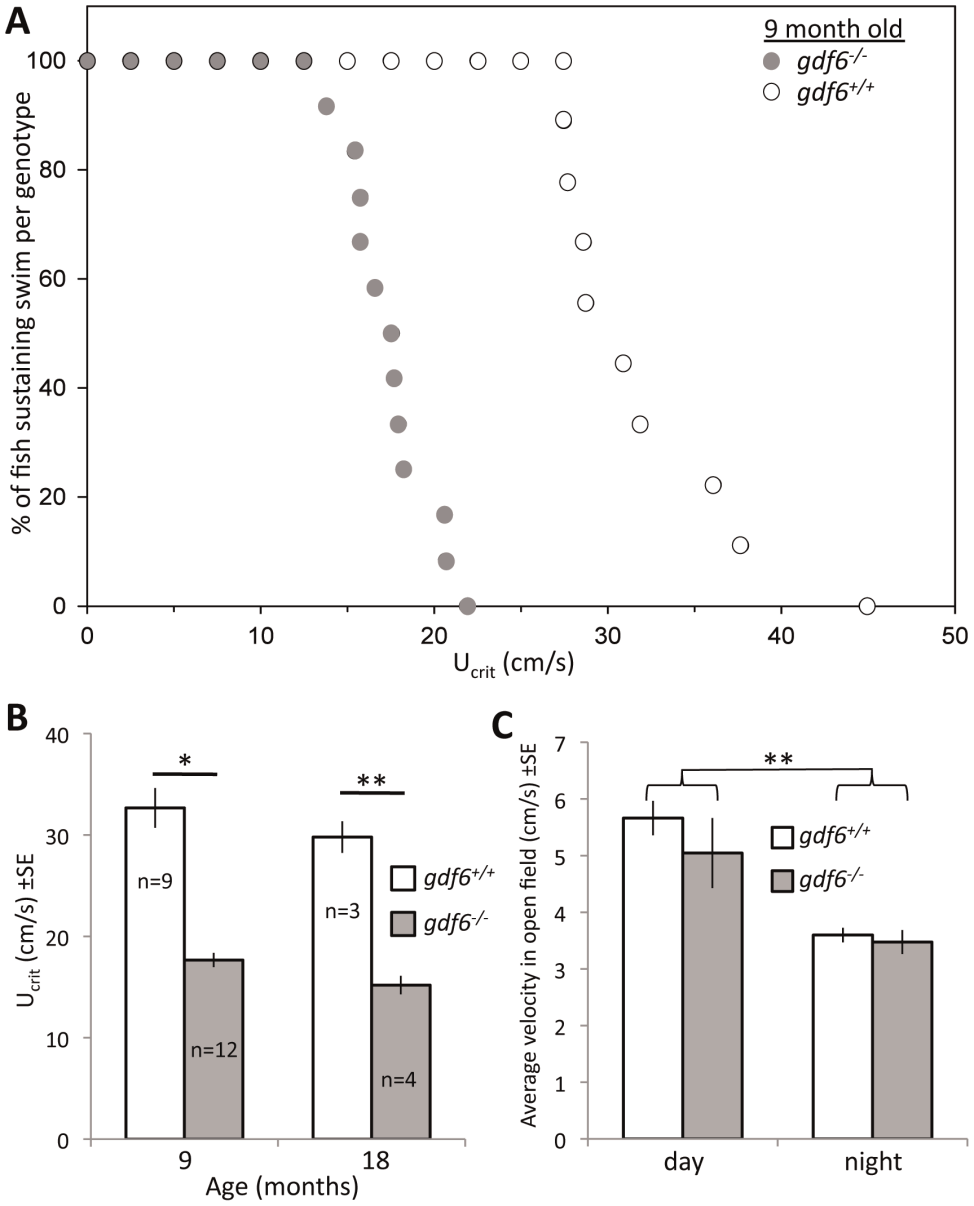
Zebrafish harboring homozygous mutations in *gdf6* exhibit decreased endurance. **A.** *gdf6^−/−^* fish have a lower endurance compared to *gdf6^+/+^* siblings as measured by increasing water velocity in a swim channel to determine the *U*_crit_ i.e. ‘critical swimming speed’ which is taken to be the highest speed that a fish can swim at for a period of several minutes before exhaustion. Each fish tested at 9 months is plotted. **B.** The same data in panel A (9 month) plotted along with 18 month old siblings. *gdf6^−/−^* fish have approximately 50% lower endurance compared to *gdf6^+/+^* siblings at each age (*p = 0.005, **p<0.01). Endurance trends towards being decreased in older fish of each genotype, but this difference is not significant. **C.** Open field tests of average swim velocity during 10 minutes of each hour through a circadian cycle, in tanks replicating lifetime husbandry conditions, show a lower mean movement at night (in either genotype, **p<0.01) but no difference between genotypes in any measure during day (p = 0.247) or night (p = 0.814).

Deficits in muscle endurance is a hallmark of ALS progression consistently observed in murine and fish models [28]. We quantified muscle endurance in *gdf6a^−/−^* and sibling fish by assessing their ability to swim against a strong, accelerating water current [35], from which we determined the critical swimming speed (*U*_crit_, the water flow velocity at which a fish can no longer maintain its position [35]; See Methods). At 9 months of age, *gdf6a^−/−^* fish were found to have substantially reduced swimming endurance, with mean *U*_crit_ values approximately half those of *gdf6^+/+^* siblings (Figure 2A,B; p = 0.005, n = 12 and 9 fish, respectively). At 18 months of age, the same significant difference was observed between genotypes (Figure 2B, p<0.0005, n = 4 and n = 3). Endurance was lower in the older fish within each genotype, though this difference between ages was not significantly different when comparing within genotypes.

The swim endurance (*U*_crit_) values in the wild type fish above are within the range of values obtained in past studies, being higher than [28], lower than [36], [37] or comparable to [38] previous results. Differences between studies are likely due to methodology, including duration of water velocity steps and flow chamber construction that can affect absolute values; thus comparisons between experimental configurations can be challenging, but the results are robust when compared within an experiment or laboratory.

An alternative interpretation of our endurance data is that fish were less able to perform the swimming test because of decreased availability of visual cues, due to microphthalmia. To rule out any confounding role for the visual deficits, swimming performance was assessed in a dark room under infrared light, which zebrafish are unable to detect (lighting conditions of the swimming endurance assay and zebrafish light sensitivity are characterized in Supplemental Figure S2). A further alternative is that the fish have decreased endurance due to decreased activity patterns in their typical husbandry conditions, perhaps predicting decreased muscle fitness. Open field tests on groups of fish were performed in conditions closely mimicking the husbandry conditions in which these fish were raised, and conducted in multiple replicates during each hour through a full circadian cycle. No significant difference in activity was found, arguing against mean activity as a confounding factor in these fish (Figure 2C, Supplemental Figure S2). The methods deployed were sensitive enough to detect lower average swim velocity in night vs. day (regardless of genotype, p<0.001), however no difference was detected between genotypes for average swim velocity during day (p = 0.247) or night (p = 0.814) (Figure 2C).

A small difference was noted between genotypes in the open field test, only during mating activity at light onset (Supplemental Figure S2), which accounted for the small non-significant decrease in overall activity of the *gdf6a^−/−^* fish. The difference observed during breeding is not relevant to the fish under consideration here, because they were raised and maintained in tanks that included sibling fish with normal eyes. When housed with normophthalmic siblings (our standard practice), *gdf6a^−/−^* fish had ample activity during breeding times and coordinately robust fecundity; this contrasts groups comprised solely of *gdf6a^−/−^* fish that had no breeding success. Thus the open field test we deployed under-represented the *gdf6a^−/−^* fish's activity during mating behaviour at light onset. In sum, the data argue against visual system deficits playing either an acute or long-term role in the muscle deficits of microphthalmic *gdf6a^−/−^* fish.

A further alternative explanation for deficits in swimming performance could be morphological changes resulting from long-term loss of *gdf6a*. Skeletal changes might be anticipated from BMP namesake functions in bone morphogenesis, from skeletal defects observed in patients with *GDF6* mutations [39]–[42] or past analysis of zebrafish *gdf6a* morphants [43]. In adult zebrafish, skeletal deficits in the axial skeleton were not observed (Figure 1, Supplemental Movie S1). Body morphology was also assessed, and no significant morphometric covariates of *U*_crit_ were observed, including condition factor (Figure S2). Caudal fin length was not different between experimental groups (One-way ANOVA F_5,31_ = 1.320 p = 0.28) and was not a significant covariant in the relationship between experimental group and swimming performance (MLR: F_1,31_ = 0.36 p = 0.55). Thus there was no evidence for a role of skeletal system malformation in the swimming deficits of *gdf6a^−/−^* zebrafish.

### gdf6a loss in adult zebrafish leads to neuromuscular disease, including disruption of neuromuscular junctions

**Figure 3:**
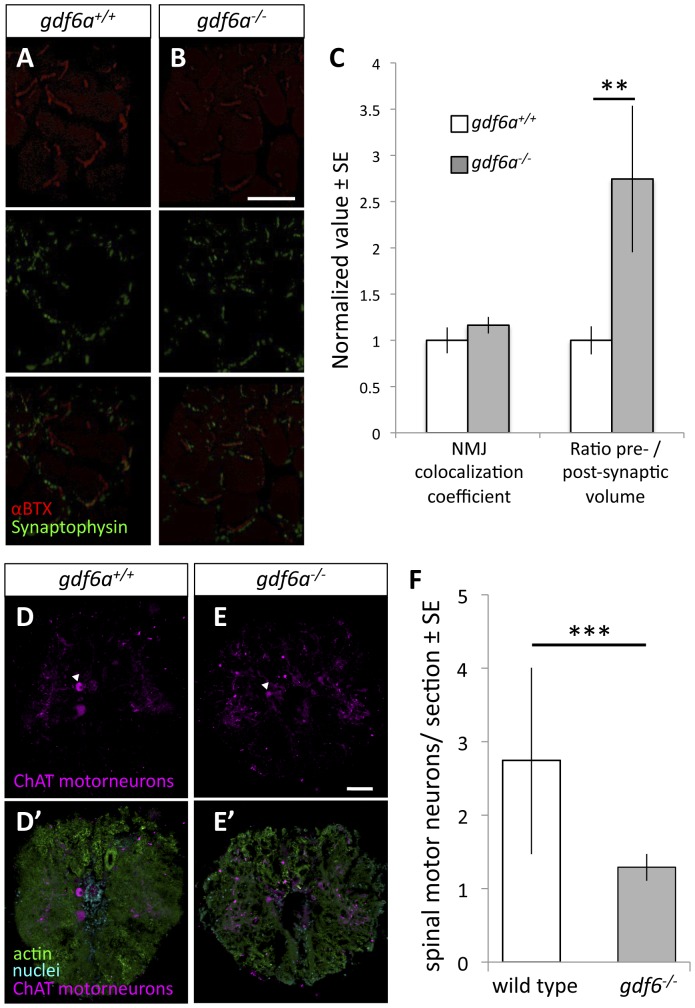
Zebrafish harboring homozygous mutations in *gdf6* exhibit altered neuromuscular junctions and fewer spinal motor neurons. **A–B.** Assessment of neuromuscular junction (NMJ) morphology including presynaptic (synaptophysin) and postsynaptic (αBTX) compartments in 9 month old *gdf6^−/−^* and *gdf6^+/+^* siblings by immunohistochemistry. **C.** ALS-like increases in motoneuron pre-synaptic volumes are observed in *gdf6^−/−^* fish when normalized to post-synaptic volumes (**p = 0.009, n = 5 fish per genotype). Coefficients of colocalization for pre- and post-synaptic compartments are not altered, as is expected in later-stage zebrafish ALS models. **D–E.** Motor neuron cell bodies were identified in cross-sections of spinal cord using immunohistochemistry against choline acetyl transferase (ChAT, e.g. arrowheads) in nine month old *gdf6^+/+^* and *gdf6^−/−^* fish (panels A and B, respectively). Bottom panels affirm motoneuron cell body identification using actin and nuclear counter-stains. **F.** *gdf6^−/−^* fish have approximately 50% the abundance of spinal motor neurons compared to sibling *gdf6^+/+^* fish. (Mann-Whitney U Test, *** p<0.001, n>50 sections from 4 fish per genotype, researcher blinded to genotype during quantification). Scale bar = 60 µm in A,B and 50 µm in D,E.

Histological hallmarks of ALS include neuromuscular deficits, comprising loss of motoneurons at the level of the spinal cord and abnormal structure of neuromuscular junctions (NMJ). Nine month old *gdf6a^−/−^* zebrafish were noted to have disrupted NMJ, including increased mean volume and greater variation of the presynaptic motoneuron compartment compared to wild type siblings (Figure 3). This presynaptic motoneuron volume, when normalized to the post-synaptic volume, was nearly 3-fold greater in *gdf6a^−/−^* mutants (p = 0.009, n = 5 fish per genotype). Co-localization of pre- and post-synaptic compartments was not significantly different based on genotype (Figure 3). Increased volume of pre-synaptic compared to post-synaptic NMJ compartments has also been observed in ALS model zebrafish overexpressing mutant SOD1 [28].

Further, *gdf6a^−/−^* fish at nine months of age were noted to have approximately 50% fewer spinal motoneurons compared to wildtype fish (p<0.001, Figure 3, n>50 sections from 4 fish per genotype), akin to changes observed in ALS model fish overexpressing mutant SOD1 [28].

### Disruption of gdf6 hastens disease progression in a zebrafish model of ALS

We reasoned that if *gdf6a* mutation hastens disease progression, or increases susceptibility to ALS, then this should be recognizable in ALS animal models lacking *gdf6a* function. We bred *gdf6a* mutants to an established zebrafish model of ALS: transgenic *os10* zebrafish that over-express zebrafish Sod1^G93R^ and develop symptoms of ALS very reminiscent of transgenic mice over-expressing human SOD1^G93A^ [28]. Breeding *gdf6a^+/−^;Tg(sod1:sod1G93R,hsp70l:DsRed)os10* fish generated six genotypes, consisting of three *gdf6a* genotypes (+/+, +/− or −/−), each with the *os10* transgene being present or absent. Herein we define ‘bigenic’ fish as those both homozygous null for *gdf6a* and possessing the *os10* transgene, i.e. *gdf6a^−/−^;Tg(sod1:sod1G93R,hsp70l:DsRed)os10*.

**Figure 4:**
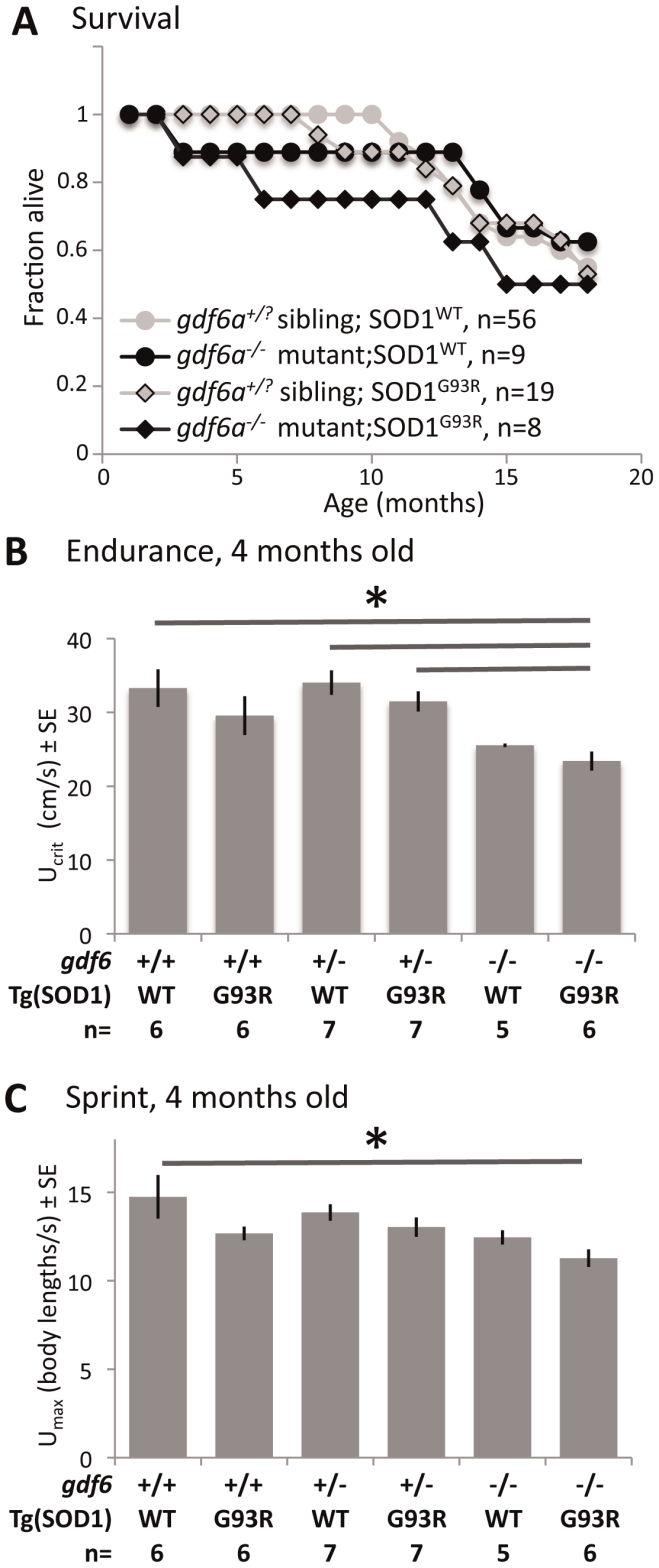
Mutations in *gdf6* sensitize SOD1∧G93R zebrafish to develop ALS-like symptoms. Six genotypes combining *gdf6^−/−^* alleles and SOD1∧G93R alleles were examined in endurance and sprint tests, which primarily measure red and white muscle respectively. The SOD1∧G93R fish mimic many aspects of ALS progression [28]. Swimming performance was measured in all six genotypes (determined by outcrosses and examining progeny) at 4.5 months of age. **A.** bigenic fish, i.e. *gdf6^−/−^* mutants expressing SOD1∧G93R, had decreased survival compared to all other genotypes, i.e. their siblings ( = *gdf6^+/+^* or *gdf6^+/−^*) with or without SOD1∧G93R. **B.** bigenic *gdf6^−/−^* mutants expressing SOD1∧G93R had significantly lower endurance compared to heterozygous siblings also expressing SOD1∧G93R, and to WT and heterozygous siblings without SOD1∧G93R. **C.** Sprint test demonstrates a significant deficit in white muscle function only when SOD1∧G93R and *gdf6^−/−^* genotypes are combined ( = 25% deficit). (ANOVA and multiple linear regression, p<0.05; sample size indicated below graph).

Similar to the observations on fish larvae with *gdf6a* lesions alone, no significant increase in axonopathy was noted in primary motoneurons when *os10* was also present in the larval genome (Supplemental Figure S1). In older fish, amongst the six genotypes examined, it was apparent that a subset of bigenic *gdf6a^−/−^;os10* fish failed to thrive. The time to reach 75% survival was approximately 6 months in bigenic ALS model zebrafish lacking normal *gdf6a* function, but more than 13 months in all other genetic combinations, including the same ALS model fish with at least partial *gdf6a* function (Figure 4A).

Muscle endurance was assessed in bigenic *gdf6a^−/−^;os10* fish and their siblings, using younger fish (4 months old) in an effort to capture any potential acceleration of disease progression. Deficits in swimming endurance attributable to *gdf6a* genotype alone were less dramatic at this young age: *gdf6a^−/−^* fish (lacking the *os10* transgene) had *U*_crit_ values approximately 75% those of their siblings but these differences were not statistically different (Figure 4B). This smaller endurance deficit in young fish contrasts the significant reductions observed at 9 and 18 months of age (where *U*_crit_ was ∼50% that of siblings, Figure 2B), providing additional support for the contention that *gdf6a* loss of function leads to neuromuscular degeneration in a progressive manner.

Similarly, young *os10* fish consistently displayed decreased endurance within each *gdf6a* genotype, similar to what was observed previously for older *os10* animals [28], though in no case did this rise to the level of statistical significance (Figure 4B). Only when the genetic lesions were combined were statistical differences attained, such that bigenic *gdf6a^−/−^;os10* fish had *U*_crit_ values approximately 65% those of wild type fish (Figure 4B, p<0.05 by MANOVA, sample size = 37, with 5–7 individuals per genotype as indicated on Figure abscissa). Thus, in young adult fish, *gdf6a* and *os10* genotypes both imposed effects on muscle endurance that were consistent with neuromuscular disease, but these deficits only reached statistical significance when both genetic lesions were combined.

Muscle strength/power was also assessed in these six genotypes, by documenting the ability of the fish to sprint against increasing velocities of water flow. This constant acceleration test was similar to the *U*_crit_ endurance test, but with water velocity increased at an accelerated rate such that fast-twitch white muscle became dominant, allowing the velocity at fatigue (*U*_max_) to be calculated [44], [45]. Neither the presence of the *os10* transgene nor loss of *gdf6a* function were individually potent in reducing swimming strength in these young fish (Figure 4C). Only when the two genetic lesions were combined was any statistically significant deficit in swimming power observed, compared to wild type fish. Thus bigenic *gdf6a^−/−^;os10* fish had *U*_max_ values approximately 75% those of wildtype fish (Figure 4C, p<0.05 by MANOVA, sample size = 37, with 5–7 individuals per genotype as indicated on the Figure).

**Figure 5:**
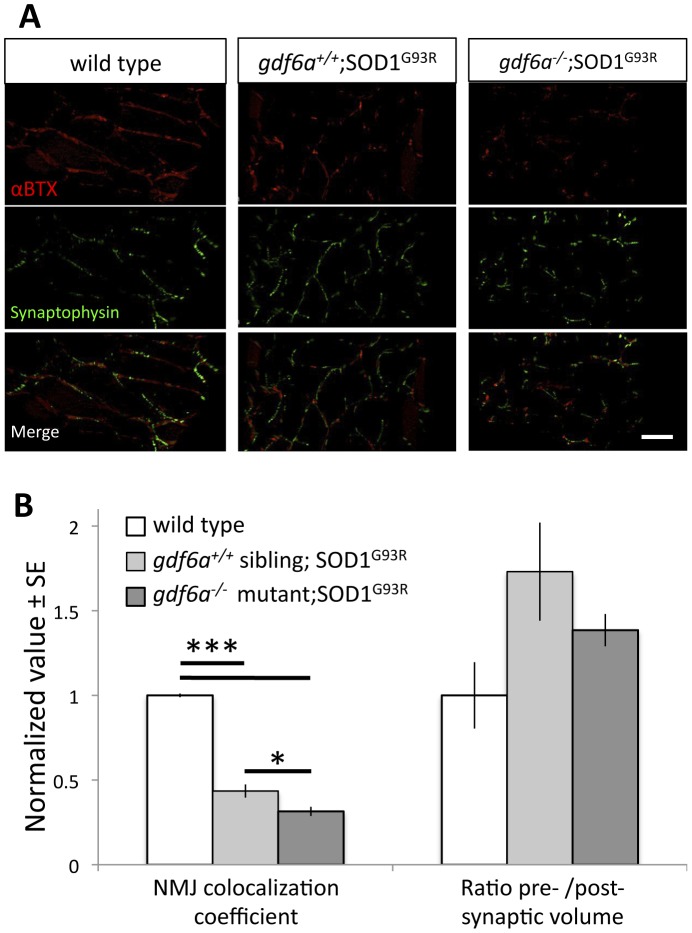
Disruption of *gdf6* function exacerbates neuromuscular junction abnormalities in ALS model zebrafish. ALS model zebrafish possess disruptions to neuromuscular junctions (NMJ), and loss of *gdf6* function exacerbates this by 7 months of age. **A.** The presynaptic junctions (labeled with synaptophysin antibody) and postsynaptic junctions (labeled with fluorescently tagged αBTX) in ALS model zebrafish expressing the mutant SOD1^G93R^ show punctate morphology, deviations in presynaptic volume and less overall colocalization compared to WT sibling junctions. Some abnormalities are exacerbated in bigenic siblings expressing the mutant SOD1^G93R^ that are also *gdf6^−/−^* (scale bar is 40 µm). **B.** Quantification of these NMJs suggests the presynaptic/postsynaptic volume ratios of SOD1^G93R^ and bigenic *gdf6^−/−^*;SOD1^G93R^ zebrafish are larger than those of WT siblings at this age, though these differences do not rise to statistical significance (Kruskall-Wallis ANOVA, p = 0.134; n = 6,4,6 for WT, SOD1^G93R^, and bigenic fish respectively). Colocalization coefficients, that measure overall colocalization of presynaptic and postsynaptic junctions, are altered in these fish. The values for SOD1^G93R^ zebrafish are significantly lower than wild type sibling values, indicating that presynapses and postsynapses overlap less, as characterized previously for this transgenic ALS model [28]. Bigenic SOD1^G93R^ zebrafish that are also *gdf6−/−* have a dramatically lower coefficient than either sets of siblings, including being 30% lower than ALS model SOD1^G93R^ fish with normal *gdf6a* (*p<0.05; ***p<0.001. Kruskall-Wallis ANOVA with pairwise comparisons).

These same bigenic *gdf6a^−/−^;os10* fish and their siblings were subsequently examined for neuromuscular junction abnormalities. Both *os10* and bigenic *gdf6a^−/−^;os10* fish at 7 months of age had increased presynaptic/postsynaptic volume ratios compared to wildtype siblings, though the differences did not reach statistical significance (Figure 5A, B. Kruskall-Wallis ANOVA p = 0.134, n = 6 WT, 4 *os10*, 6 *gdf6a^−/−^;os10*). Further, NMJs in bigenic *gdf6a^−/−^;os10* had significantly decreased overlap of synaptic compartments compared to either *os10* or wildtype siblings (Figure 5B) (Kruskall-Wallis ANOVA, p<0.001). Mander's colocalization coefficients for the postsynaptic compartment were significantly lower for bigenic *gdf6a^−/−^;os10* fish, implying loss of synaptic connectivity because postsynaptic compartments were less colocalized with presynaptic compartments despite the increase in presynaptic size (Figure 5B) (Kruskall-Wallis ANOVA, p<0.05). Thus the *gdf6a^−/−^* mutation does not alter co-localization of NMJ pre- and post-synaptic compartments on its own (Figure 3C), but it further exacerbates the ALS-like NMJ abnormalities observed in bigenic fish expressing overexpressing mutant SOD1.

Overall, loss of Gdf6 function led to an acceleration of disease progression in this ALS animal model, as measured by longevity, integrity of neuromuscuslar junctions, muscle endurance and muscle strength. These data are in good accordance with the hypothesis that deficits at the *GDF6* locus can sensitize animals to onset and/or progression of neuromuscular degeneration akin to ALS.

## Discussion

Zebrafish harboring homozygous null mutations in a *GDF6* homologue were found to be viable, fecund and robust into adulthood, though exhibiting somewhat reduced longevity. Homozygous *gdf6a^−/−^* fish had progressively severe deficits in endurance, loss of spinal motoneurons and disrupted neuromuscular junctions compared to the wild type siblings, very much akin to ALS model zebrafish. Furthermore, progression of neuromuscular disease in ALS model zebrafish was accelerated when this homologue of *GDF6* was mutated: older zebrafish expressing mutant SOD1 exhibit ALS-like symptoms, but young fish only exhibited significant ALS-like neuromuscular deficits when on a *gdf6a^−/−^* background.

Regarding ALS and other neuromuscular diseases, support for a BMPs' relevance is continuing to be revealed by genetic studies in *Drosophila*, including that a homologue of BMPs and homologues of BMP receptors are each required for normal NMJ development in flies [1], [4], [46], [47], but suggestions of such functions for BMP pathway components rarely extend to vertebrates. Within vertebrates, BMP receptor type II has recently been localized to mouse NMJs, and one of its ligands BMP4 was found in close proximity [48]. BMP4, BMP5 and their receptors have also been shown to have a role at some synapses in the CNS [49]. A role for BMPs in the vertebrate neuromuscular system has more frequently been suggested following motoneuron or spinal cord injury [50]. Recent work has also demonstrated a role for proper trafficking, including that of BMP receptors, in primary motor axon outgrowth during zebrafish development [30], instigating a search for which component of the BMP gene family might underpin this effect. A potential example may be GDF15, a member of the BMP subfamily with divergent properties and typically associated with macrophage recruitment and inflammation, in that GDF15 disruption causes loss of motoneurons in mice [51]. A link between BMP signaling and neuromuscular disease is suggested by the observation of phosphoSMAD proteins (downstream signaling components of TGFβ/BMP/GDF signaling) being enriched in pathological ALS inclusions [31]–[33], [52]. Human genetics suggests that loci associated with hereditary spastic paraplegia, including ATLASTIN, can regulate BMP receptor trafficking and effect neuromuscular disease [30], [50]. Despite this substantial level of interest, we are not aware of previously published data from vertebrate experiments or human genetics directly linking BMP/GDF gene family members (ligands or receptors) with ALS or any neuromuscular disease.

A substantial proportion of ALS susceptibility remains unexplained with respect to human genetics. Analyses herein support *GDF6*, a member of the BMP family, as worthy of further consideration. A role for *GDF6* homologues in progressive, late-onset neuromuscular disease was supported herein by muscle physiology, histopathology and behavioural assessment of zebrafish carrying mutations in *gdf6a*. Further, *gdf6a* loss of function accelerated disease progression in a zebrafish model of ALS. The synthesis of this data combines with recent reports of a role for GDF6 in congenital and late-onset photoreceptor degenerations [34], [53] to compel a role for GDF6 as a modifier gene in the etiology of disparate neuropathies.

### BMPs in neurodegenerative disease

Inappropriate growth factor expression and signaling have been linked to inflammation and apoptosis in other neural diseases such as Alzheimer's, Parkinson's and, in one instance, Creutzfeldt-Jakob Disease, which are characterized by inappropriate accumulation of misfolded proteins, leading to neuron death via a multitude of proposed pathological pathways [54]–[56]. TGFβ1 and TGFβ2 expression are high in brains afflicted with Alzheimer's, causing inflammation, astrogliosis and neuron death through such pathways as NADPH oxidase-induced oxidative stress and caspases [57], [58]. Xenopus TGFβ1, a member of the same gene superfamily as GDF6, is released from Schwann cells and can promote synaptogenesis at NMJ [59]. TGFβ1 is an effector of motoneuron survival (refs in [60]) and has been proposed as a mediator of increased ALS susceptibility in athletes abusing anabolic steroids [60]. Based on these similarities, it is plausible that GDF6 dysfunction could contribute to any of these pathways as they play out in ALS. Based on our data, we believe the expansion of ALS studies to the investigation of TGFβ signaling is warranted, beginning with *GDF6*.

### Therapeutic implications

Application of BMPs as treatment has been investigated previously [50], especially following injury, suggesting that further knowledge of *GDF6* function could lead to strategies that replace BMP function. A number of TGFβ superfamily members are neuroprotective, such as BMP7 in cerebral ischemia and GDF5 in Parkinson's disease [61], [62]. GDF15 plays a role in the pathology of motoneuron loss, but is also required to effect the therapeutic effects of GDNF [51], [63], illustrating how these signaling factors are able to participate in either neuroprotection or degeneration.

Therapeutic potential also exists in small molecules such as aminopropyl carbazoles and their derivatives. For example it has recently been demonstrated that P7C3 can ameliorate phenotypes in zebrafish *gdf6a* mutants [34], and P7C3 has also recently been shown to be efficacious in mouse models of ALS and Parkinsons Disease [64], [65]. Efficacy of P7C3 seems to be driven by a combination of pro-neural and neuroprotective signals, and identifying the mechanisms that underpin this could be aided by zebrafish models and/or by investigation of its etiological effects in diverse neurodegenerations.

Finally, *GDF6* may represent an entry-point into unexplored pathogenic mechanisms of vertebrate neuromuscular degeneration. In cases where such mechanisms have been identified, the etiology of ALS can be associated with a variety of cellular mechanisms. Ferraiuolo and colleagues [66] review these to include oxidative stress, RNA processing, endosomal trafficking, protein degradation, cytoskeletal integrity and glutamate excitotoxicity. It is conspicuous that, despite being a member of the BMP family with diverse functions, *GDF6* is not currently associated with any of these etiological categories.

Three mechanisms whereby BMP ligands and receptors affect NMJs can be suggested as potential routes to explore in the zebrafish system. First, retrograde signaling of BMP ligand from the muscle to the NMJ, and retrograde transport of receptors to activate transcription in the nucleus of motoneurons is important for motoneuron survival in *Drosophila* [4], [50]. Second, BMP in *Drosophila* is required for stabilizing the NMJ through non-canonical signaling via LIM Kinase domain 1 [4], [50]. Third, vertebrate BMP ligands have recently been revealed to bind Agrin [67], and thus directly affect the clustering of acetylcholine receptors during formation and stabilization of the NMJ. In support of the latter we found that the NMJ morphology, as measured by α-bungarotoxin labelling of acetylcholine receptors, was disrupted in *gdf6a^−/−^* mutants (Figures 3, 5), suggesting one priority for future analysis.

### Conclusion

In summary, past conclusions regarding a role for *Drosophila* BMP ligands in effecting NMJ can be extended to vertebrates, supporting conservation of mechanisms that may be of substantial import to degenerative neuromuscular diseases. Zebrafish present a tractable system for assessing the role of BMP/GDF ligands (data herein) and their receptors [30] with respect to neuromuscular disease. The data also synergize to argue for further analysis of *GDF6* and other *BMP* genes as modifier loci, affecting susceptibility to ALS and perhaps a broader suite of neurodegenerative diseases. Further efforts to assess the role of vertebrate BMPs in motoneuron disease are warranted.

## Methods

### Ethics statement

All fish husbandry and procedures were completed with approval of the University of Alberta Animal Care and Use Committee: Biosciences, in accordance with the Canadian Council on Animal Care.

### Zebrafish husbandry and establishment of mutants and transgenic crosses

Zebrafish bearing *gdf6a^s327/s327^* (also known as *radar^ s327/s327^* [13], Zfin ID ZDB-ALT-050617-10), referred to here as *gdf6^−/−^*, previously identified as larval lethal, were recently acquired as viable adults through breeding of heterozygous mutants, preceded by backcrosses onto an AB background [34]. Homozygous *gdf6a^s327/s327^* larvae were identified by the presence of microphthalmia at 3dpf and were raised separately from their siblings. Mutants and siblings were confirmed in several cases by genotyping from finclip derived DNA at adulthood (using primers 5′-TGTGAGACACGGCTCCACTT-3′ and 5′-GCAGGACGAGAGCTTACCAA-3′) and/or by examining the Mendelian frequency with which their progeny exhibited microphthalmia. At 9 and 18 months, Gdf6a^s327/s327^ adults and WT siblings underwent a swim channel assay and were subsequently sacrificed for NMJ and spinal cord immunocytochemistry.

ALS model zebrafish, expressing mutant SOD1^G93R^ under control of the zebrafish *sod1* promoter [28], were generously provided by Christine E. Beattie. The fish, *Tg(sod1:sod1G93R,hsp70l:DsRed)os10* (ZFin ID: ZDB-GENO-101006-6), herein referred to as *os10*, can be readily identified from their expression of DsRed under a promoter that is independent of the *sod1* expression cassette, but linked immediately contiguous to it in the construct. The *os10* line was crossed to *gdf6a^s327/s327^* fish, and screened for the presence of *os10* transgene following heat shock at 2dpf [28]; the resultant compound heterozygous fish were inbred to acquire the combinations of *gdf6a^s327^* and *os10* in the various combinations reported.

Zebrafish husbandry and breeding used standard methods [68], with 14∶10 L∶D light cycle, 1300±100 µS conductivity at 28±1°C water temperature. Fish were fed a diet dominated by brine shrimp and trout chow.

### Assessing primary motoneuron axonopathy in embryonic zebrafish

Zebrafish embryos were scored for abnormal primary motoneuron axonopathy, as previously described [27], in transgenic animals where primary motoneurons abundantly express GFP under control of the promoter *mnx1* (also known as *hb9*, Zfin ID: ZDB-GENO-080606-250). Breeding created *gdf6a^+/−^;Tg(mnx1:GFP)ml2* fish. These were bred to *gdf6a^+/−^;Tg(sod1:sod1G93R,hsp70l:DsRed)os10* fish described above to generate genotypes including *gdf6a^−/−^* fish and siblings with labelled motoneurons and/or expressing mutant Sod1^G93R^.

At 30hpf, larvae were visualized under a Leica MZ16F fluorescent stereomicroscope and axon branching was scored while the researcher was blind to genotype. As per established methods [27], branching of the GFP positive motoneurons was considered abnormal if it initiated at or above the ventral aspect of the notochord. Branching that was observed below this point was considered normal. The yolk sac extension was used as a landmark, and only the ten motoneurons immediately above it were scored for defects (Figure S1). Subsequent to quantifying its axonopathy, each of the larvae was reared individually in 24 well plates until they were old enough to assess genotypes based on microphthalmia, and heat-shocked for one hour prior to screening for DsRed expression associated with *os10*. To assess the sensitivity of the axonopathy assay in our hands, *Tg(mnx1:GFP)ml2* fish were injected with 900 pg of mRNA encoding wildtype or A4V human SOD1. mRNAs were synthesized and delivered as per previous methods [69]. Axonopathy scores per fish were analyzed using a Kruskall-Wallis ANOVA followed by a pairwise Mann-Whitney U-test, calculated in SYSTAT 12. All p-values reported are two-tailed and significance was set at p<0.05.

### Adult fish - assessing endurance in swim channel assay and activity in open field test

Individual fish were tested for their critical sustained swimming (*U*_crit_), which was the maximum velocity at which they could swim for a set period in a 10 L modified Brett-style swim tunnel (Loligo Systems, DK). Fish were placed in the swim tunnel under blackout hoods with infrared lighting (see below for characterization of lighting) and allowed to acclimate for 30 minutes to a low velocity current (5 cm/s). The flow rate was then increased every 10 minutes by 4 cm/s. Fish were monitored via infrared camera (SAV-CD120; Matco, QE) and Elgato video capture software (Elgato Systems, CA), and were considered to have reached maximum velocity when they were no longer able to maintain position in the current and fell into a screen at the back of the swim chamber. After fish fatigued the test was halted and the fish removed following a cool-down swimming period of 10 minutes at low velocity. The four month old fish were allowed to recover in the chamber at 5 cm/s for 45-minutes before completing a second test with shortened, one-minute steps to determine their maximum sprint swimming speed (*U*_max_). The U_crit_ and U_max_ values were calculated using the Brett formula [70]. All fish were measured for morphological traits that are potential covariates of swimming endurance [36], [38]. Morphology measurements included: head length, muscle length, standard length, fork length, total length, body height, body width, caudal peduncle, and mass. Condition factor was determined using the standard formula from mass (g) and body length (BL; mm), calculated as 10^5^·mass/BL^3^.

Open field tests on zebrafish were carried out on groups of three fish per tank under conditions nearly identical to their normal husbandry conditions regarding temperature, water chemistry, water flow rates and lighting (except that IR lights were used to enable video recording in the dark). Fish were transferred from their housing tanks to test tanks of the same dimensions and were acclimated for one hour prior to the start of testing (N = 6 tanks with 3 fish each). Zebrafish motion was then recorded from above for 10-minutes every hour for 24-hours, using IR-lit cameras (as above) and video surveillance software (Visio-soft; Matco, QE). Average swimming speeds were determined from video (15 measures per second) for each fish using behavioural analysis software (Ethovision XT8.5; Noldus, NL). Infrared lighting was characterized using a calibrated spectrophotometer (USB4000-UV-VIS Ocean Optics, Dunedin FL) connected to a 455 micron optical fiber (QP450-2-XSR, Ocean Optics) held at the approximate position that fish maintained during trials. Spectra were recorded to Spectra Suite software (Ocean Optics).

Differences in the day- and night-time swimming speed between *gdf6^−/−^* and wild-type fish were assessed using a two-way repeated measures analyses of variance with a Holm-Sidak post-hoc test. Differences in U_max_ (BL/s) and U_crit_ (cm/s) between genotypes were analyzed using one-way ANOVA's with Holm-Sidak post-hoc tests. Holm-Sidak adjusted p-values were reported. All swimming performance and open field test data were log transformed to achieve normality. All data were tested for normality (Shapiro-Wilk) and equal variance (Levene's test) prior to the use of parametric statistics. Multiple linear regression analyses were also performed using standard body length and genotype as explanatory variables of swimming performance to ensure that the detected differences were independent of fish size. The statistical analysis of swimming performance and activity was conducted using SPSS 20.0 (SPSS IBM, Chicago, IL) and Sigmaplot 11 (Systat Software, CA).

### Adult zebrafish tissue preparation and immunocytochemistry

To assess neuromuscular junctions (NMJ), muscles from adult zebrafish were removed, cut transversely into quarters (anterior to posterior) and fixed in 4% paraformaldehyde/0.1 M PO_4_ for 24 hours at 4°C. Samples were prepared for cryosectioning as per previous methods [71] by washing thrice in 5% sucrose/0.1 M PO_4_ (20 minutes), once in 12.5% sucrose/0.1 M PO_4_ (30 minutes), and overnight in 20% sucrose/0.1 M PO_4_. Samples were embedded and frozen in OCT media (Tissue-Tek 62550-12). 16 µm sections were serially placed on Superfrost Plus microscope slides (Fisherbrand 12550-15). All sections used in immunocytochemistry were of the second-most anterior quarter of muscle.

NMJs were visualized by labeling pre-synaptic terminals and post-synaptic terminals as reported previously [28]. Briefly, slides were blocked for 60 minutes in 10% NGS/PBSTw and incubated in 1∶50 rabbit anti-synaptophysin (Invitrogen 180130) in 2% NGS/PBSTw overnight at 4°C. Slides were washed with PBSTw and incubated in 1∶500 AlexaFluor 488 chicken anti-rabbit (Invitrogen A21441)/1∶100 AlexaFluor 555-tagged α-bungarotoxin (Invitrogen B35451)/2% NGS/PBSTw overnight at 4°C. Sections were viewed with a Zeiss LSM 700 confocal mounted on a Zeiss Axio Observer.Z1 microscope and imaged with ZEN 2010 (version 6.0) software (Carl Zeiss MicroImaging).

To quantify motoneuron abundance, spinal cords were dissected whole and fixed in 4% paraformaldehyde/0.1 M PO_4_ for 24 hours at 4°C. The spinal cords were then prepared for cryosectioning as previously described. Transverse sections (20 µm) of each sample were serially placed on 6 Superfrost Plus microscope slides. Motor neurons were labeled as previously described [28]. Briefly, slides were incubated in 0.003% H_2_O_2_/PBS for 20 minutes, blocked for 1 hour in 5% normal donkey serum (NDS)/1% DMSO/PBSTw, washed, and incubated in 1∶100 goat anti-ChAT (Chemicon AB 144P)/2% NDS/1% DMSO/PBSTw for 3 days at 4°C. Slides were rinsed and washed in PBSTw, and incubated in 1∶500 Alexafluor 555 or Alexafluor 488 donkey anti-goat (Invitrogen A-21432 and A-11055)/2% NDS/1% DMSO/PBSTw overnight at 4°C. Actin was labelled using phalloidin conjugated to AlexaFluor-488 (Invitrogen #A12379). Nuclei were stained with TO-PRO-3 (Invitrogen, #T3605). After three 30-minute washes with PBSTw, slides were coverslipped and imaged. Sections were viewed with a Zeiss LSM 700 confocal mounted on a Zeiss Axio Observer.Z1 microscope and imaged with ZEN 2010 (version 6.0) software (Carl Zeiss MicroImaging). Powerpoint 2008 for Mac (Microsoft) was used to assemble figures following being merged and/or linearly manipulated for brightness and contrast in Photoshop CS3, Zen confocal software (Zeiss), and/or Imaris ×64 7.4.0 (Bitplane).

### Histological analysis

NMJ volumes were measured using the voxel counter plugin for ImageJ 1.45 (Wayne Rasband, National Institutes of Health; http://rsbweb.nih.gov/ij/index.html) and colocalization was analyzed in Imaris ×64 (version 7.4.0, Bitplane). Researcher was blinded to genotype prior to image analysis. Values were normalized to WT values and statistical analysis was performed with Kruskall-Wallis ANOVA (volume and colocalization measurements) and Mann-Whitney U test (colocalization measurements) on SYSTAT 12.

Motoneurons in spinal cord cross-sections were quantified as per established methods [28] by averaging the number of motoneuron cell bodies, identified as ChAT-positive objects greater than 10 µm, per section. Researcher was blinded to genotype during quantification and analysis. Statistical analysis was performed with Kruskall-Wallis ANOVA in SYSTAT 12.

### Clearing and staining of skeletal elements

Clearing and staining of cartilage and bone was performed using established protocols [72]. Briefly, adult fish were fixed whole in 4% paraformaldehyde overnight, rinsed in distilled water and preserved step-wise in 30%, 70% and 95% ethanol. The fish were then placed in alcian blue stain (cartilage) (Sigma-Aldrich A5268) in 30% acetic acid/70% EtOH for 6 hours and placed in saturated sodium borate overnight. Depigmentation was performed using 15% peroxide/85% KOH for 25–50 minutes or until pigment was removed. Fish were partially cleared in 30% saturated sodium borate with 1∶250 tissue culture grade trypsin (VWR, CA97061-708)(1/16 teaspoon/30 mL). Bones were stained using a 1∶1000 dilution of stock alizarin red dye (Sigma-Aldrich A5533) in 1% KOH for 1 hour, or until sufficiently pink. Afterward, fish were fully cleared in trypsin solution and preserved step-wise in 30% and 70% glycerin/1% KOH. Fishes were finally stored and imaged in 100% glycerin.

### Imaging skeletal elements in zebrafish by microCT

Intact fish were fixed in 4% paraformaldehyde/5% sucrose in 0.1 M PO_4_ buffer for 24 hours and washed in 0.1 M PO_4_ pH 7.4. buffer 3-times for 20 minutes to remove fixative. Fish were incubated overnight in Lugol solution (Sigma-Aldrich; No. L-6146) with gentle agitation followed by three 20-minute baths in 0.1 M PO_4_ buffer. Tissue was dehydrated in a graded ethanol series and kept at −20°C. To orient the specimen appropriately for scanning, it was partially embedded vertically in 2% agarose, caudal fin down, with the top half of a 15 mL Falcon tube serving as a mold. Imaging was performed on a SkyScan1174 (Bruker; Kontich, BE) compact Micro CT (50 kV x-ray source and inbuilt 1.3 MP cooled x-ray camera). Raw data obtained from the scanner was initially reconstructed using NRecon v. 1.6.6 (Bruker; Kontich, BE) and further processed to optimize viewing of structures of interest using Osirix v. 5.0.2 (Open-source DICOM Viewer). 360° rotating specimen videos were produced using Osirix v. 5.0.2 for viewing.

## Supporting Information

Figure S1 **In larval zebrafish, mutations in** ***gdf6a*** **do not appreciably sensitize SOD1∧G93R zebrafish to develop ALS-like symptoms.** Four genotypes combining *Gdf6^−/−^* alleles and SOD1∧G93R alleles were examined in *Tg(HB9:eGFP)* zebrafish expressing GFP in the axons of primary motor neurons (PMN), or via immunohistochemistry. **A.** Bracket indicates position of axons quantified, magnified in B,C. **B.** Normal primary motor axons. **C.** An example of an abnormal PMN axon (arrow). **D.** Quantification of primary motor axons in 30 hour post-fertilization embryosshow no difference based on *gdf6a* genotype; Thus effects are not developmental, and accord with a late-onset phenotype. Results indicated no significant effect of Gdf6 on the presence of axonopathies (p≥0.315, n≥5 larvae per genotype), although the highest rate of PMN axon abnormalities were observed in *gdf6a^−/−^;SOD1^+/G93R^* larvae.
(TIF)

Figure S2 **Eliminating alternate hypotheses that might account for differences in swimming behaviour between** ***gdf6a^−/−^*** **zebrafish and their siblings.** Our overall conclusion is that *gdf6a* mutants have deficits in endurance due to a progressive loss of spinal motor neurons and disrupted neuromuscular junctions. Alternative explanations for these fish having reduced endurance are eliminated here. **A. Body morphology was not significantly different based on genotype.** Condition factor was determined using the standard formula from mass (g) and body length (BL, in mm), calculated as 100000Xmass/BL^3^. Condition factor did not vary based on genotype. Sample sizes (number of fish) indicated at the bottom of graph. **B. Acute deficits in vision cannot account for differences between microphthalmic** ***gdf6a^−/−^*** **fish and their wildtype siblings.** Infrared lighting conditions during behavioural tracking of zebrafish excludes a role for visual dysfunction in the assays of fish activity, power or endurance. Grey trace indicates photons available to fish during recording sessions. Arrows annotate the maximal wavelength of sensitivity of photoreceptors in zebrafish [73]: rod photoreceptors (grey) and cone photoreceptors (coloured to indicate spectral sensitivity, magenta for ultraviolet-sensitive cone, and blue-, green- and red-sensitive cones are indicated by the cognate colour) are documented. In sum, the infrared conditions used prevented vision from impacting behaviour of wild type or mutant fish the during tracking of fish movement in the open field test, endurance tests or sprint tests. **C.** ***gdf6a^−/−^*** **zebrafish in open field test shows no significant difference in average movement compared to wild type siblings.** Fish movement tracked over 24 hours, bar below abscissa indicates lights on and off. Mutant fish had near-normal activity levels throughout the circadian cycle. A lower average movement in mutants is noted immediately after the lights turn on (0800–0900 h, compared to siblings), though this difference is not expected to occur in normal husbandry conditions: The data herein tracked groups of mutant fish, or groups of sibling fish, maintained in separate tanks (because automated video tracking of individuals in mixed populations was unreliable), whereas in normal husbandry conditions the microphthalmic *gdf6a^−/−^* mutants were housed in the same tanks as their siblings. This is impactful, because breeding is cued by lights turning on, and microphthalmic fish had increased movement during breeding (and bred successfully) only if housed with normal fish. If all fish in a tank are microphthalmic *gdf6a^−/−^*, they fail to exhibit breeding behaviour and thus have less vigorous movement. Because our *gdf6a^−/−^* fish were raised in mixed populations with normophthalmic siblings, they likely had near-normal movement activity when the lights were automatically turned on each morning. This data is summarized in Figure 4C, concluding no significant difference in total movement between genotypes.
(TIF)

Movie S1 ***gdf6^−/−^*** **fish have normal skeletal morphology compared to wildtype siblings.** See Figure 1 regarding scale bars.
(MOV)

## References

[1] McCabe BD, Marques G, Haghighi AP, Fetter RD, Crotty ML, et al (2003) The BMP homolog Gbb provides a retrograde signal that regulates synaptic growth at the Drosophila neuromuscular junction. Neuron 39: 241–254. 12873382 10.1016/s0896-6273(03)00426-4

[2] Baines RA (2004) Synaptic strengthening mediated by bone morphogenetic protein-dependent retrograde signaling in the Drosophila CNS. J Neurosci 24: 6904–6911. 15295025 10.1523/JNEUROSCI.1978-04.2004 PMC6729602

[3] Kahlem P, Newfeld SJ (2009) Informatics approaches to understanding TGFbeta pathway regulation. Development 136: 3729–3740. 19855015 10.1242/dev.030320 PMC2766340

[4] Bayat V, Jaiswal M, Bellen HJ (2011) The BMP signaling pathway at the Drosophila neuromuscular junction and its links to neurodegenerative diseases. Curr Opin Neurobiol 21: 182–188. 20832291 10.1016/j.conb.2010.08.014 PMC3095363

[5] Ruschke K, Hiepen C, Becker J, Knaus P (2012) BMPs are mediators in tissue crosstalk of the regenerating musculoskeletal system. Cell Tissue Res 347: 521–544. 22327483 10.1007/s00441-011-1283-6

[6] Marques G (2005) Morphogens and synaptogenesis in Drosophila. J Neurobiol 64: 417–434. 16041756 10.1002/neu.20165

[7] Marques G, Haerry TE, Crotty ML, Xue M, Zhang B, et al (2003) Retrograde Gbb signaling through the Bmp type 2 receptor wishful thinking regulates systemic FMRFa expression in Drosophila. Development 130: 5457–5470. 14507784 10.1242/dev.00772

[8] Eaton BA, Davis GW (2005) LIM Kinase1 controls synaptic stability downstream of the type II BMP receptor. Neuron 47: 695–708. 16129399 10.1016/j.neuron.2005.08.010

[9] Dion PA, Daoud H, Rouleau GA (2009) Genetics of motor neuron disorders: new insights into pathogenic mechanisms. Nat Rev Genet 10: 769–782. 19823194 10.1038/nrg2680

[10] Nishimura AL, Mitne-Neto M, Silva HC, Richieri-Costa A, Middleton S, et al (2004) A mutation in the vesicle-trafficking protein VAPB causes late-onset spinal muscular atrophy and amyotrophic lateral sclerosis. Am J Hum Genet 75: 822–831. 15372378 10.1086/425287 PMC1182111

[11] Ratnaparkhi A, Lawless GM, Schweizer FE, Golshani P, Jackson GR (2008) A Drosophila model of ALS: human ALS-associated mutation in VAP33A suggests a dominant negative mechanism. Plos One 3: e2334. 18523548 10.1371/journal.pone.0002334 PMC2390852

[12] Graff JM (1997) Embryonic patterning: to BMP or not to BMP, that is the question. Cell 89: 171–174. 9108472 10.1016/s0092-8674(00)80196-8

[13] Gosse NJ, Baier H (2009) An essential role for Radar (Gdf6a) in inducing dorsal fate in the zebrafish retina. Proc Natl Acad Sci U S A 106: 2236–2241. 19164594 10.1073/pnas.0803202106 PMC2650138

[14] French CR, Erickson T, French DV, Pilgrim DB, Waskiewicz AJ (2009) Gdf6a is required for the initiation of dorsal-ventral retinal patterning and lens development. Dev Biol 333: 37–47. 19545559 10.1016/j.ydbio.2009.06.018

[15] Chang C, Hemmati-Brivanlou A (1999) Xenopus GDF6, a new antagonist of noggin and a partner of BMPs. Development 126: 3347–3357. 10393114 10.1242/dev.126.15.3347

[16] Sasagawa S, Takabatake T, Takabatake Y, Muramatsu T, Takeshima K (2002) Axes establishment during eye morphogenesis in Xenopus by coordinate and antagonistic actions of BMP4, Shh, and RA. Genesis 33: 86–96. 12112877 10.1002/gene.10095

[17] Rissi M, Wittbrodt J, Delot E, Naegeli M, Rosa FM (1995) Zebrafish Radar: a new member of the TGF-beta superfamily defines dorsal regions of the neural plate and the embryonic retina. Mech Dev 49: 223–234. 7734395 10.1016/0925-4773(94)00320-m

[18] Miyazono K, Maeda S, Imamura T (2005) BMP receptor signaling: transcriptional targets, regulation of signals, and signaling cross-talk. Cytokine Growth Factor Rev 16: 251–263. 15871923 10.1016/j.cytogfr.2005.01.009

[19] Pokrishevsky E, Grad LI, Yousefi M, Wang J, Mackenzie IR, et al (2012) Aberrant localization of FUS and TDP43 is associated with misfolding of SOD1 in amyotrophic lateral sclerosis. Plos One 7: e35050. 22493728 10.1371/journal.pone.0035050 PMC3320864

[20] Hewamadduma CA, Grierson AJ, Ma TP, Pan L, Moens CB, et al (2013) Tardbpl splicing rescues motor neuron and axonal development in a mutant tardbp zebrafish. Hum Mol Genet 22: 2376–2386. 23427147 10.1093/hmg/ddt082 PMC3658164

[21] Kabashi E, Bercier V, Lissouba A, Liao M, Brustein E, et al (2011) FUS and TARDBP but not SOD1 interact in genetic models of amyotrophic lateral sclerosis. PLoS Genet 7: e1002214. 21829392 10.1371/journal.pgen.1002214 PMC3150442

[22] Laird AS, Van Hoecke A, De Muynck L, Timmers M, Van den Bosch L, et al (2010) Progranulin is neurotrophic in vivo and protects against a mutant TDP-43 induced axonopathy. Plos One 5: e13368. 20967127 10.1371/journal.pone.0013368 PMC2954192

[23] Metz GA, Merkler D, Dietz V, Schwab ME, Fouad K (2000) Efficient testing of motor function in spinal cord injured rats. Brain Res 883: 165–177. 11074045 10.1016/s0006-8993(00)02778-5

[24] Schmid B, Hruscha A, Hogl S, Banzhaf-Strathmann J, Strecker K, et al (2013) Loss of ALS-associated TDP-43 in zebrafish causes muscle degeneration, vascular dysfunction, and reduced motor neuron axon outgrowth. Proc Natl Acad Sci U S A 110: 4986–4991. 23457265 10.1073/pnas.1218311110 PMC3612625

[25] Vaccaro A, Patten SA, Ciura S, Maios C, Therrien M, et al (2012) Methylene blue protects against TDP-43 and FUS neuronal toxicity in C. elegans and D. rerio. Plos One 7: e42117. 22848727 10.1371/journal.pone.0042117 PMC3407135

[26] Van Hoecke A, Schoonaert L, Lemmens R, Timmers M, Staats KA, et al (2012) EPHA4 is a disease modifier of amyotrophic lateral sclerosis in animal models and in humans. Nat Med 18: 1418–1422. 22922411 10.1038/nm.2901

[27] Lemmens R, Van Hoecke A, Hersmus N, Geelen V, D'Hollander I, et al (2007) Overexpression of mutant superoxide dismutase 1 causes a motor axonopathy in the zebrafish. Hum Mol Genet 16: 2359–2365. 17636250 10.1093/hmg/ddm193

[28] Ramesh T, Lyon AN, Pineda RH, Wang C, Janssen PM, et al (2010) A genetic model of amyotrophic lateral sclerosis in zebrafish displays phenotypic hallmarks of motoneuron disease. Dis Model Mech 3: 652–662. 20504969 10.1242/dmm.005538 PMC2931540

[29] McGown A, McDearmid JR, Panagiotaki N, Tong H, Al Mashhadi S, et al (2013) Early interneuron dysfunction in ALS: insights from a mutant sod1 zebrafish model. Ann Neurol 73: 246–258. 23281025 10.1002/ana.23780 PMC3608830

[30] Fassier C, Hutt JA, Scholpp S, Lumsden A, Giros B, et al (2010) Zebrafish atlastin controls motility and spinal motor axon architecture via inhibition of the BMP pathway. Nat Neurosci 13: 1380–1387. 20935645 10.1038/nn.2662

[31] Nakamura M, Ito H, Wate R, Nakano S, Hirano A, et al (2008) Phosphorylated Smad2/3 immunoreactivity in sporadic and familial amyotrophic lateral sclerosis and its mouse model. Acta Neuropathol 115: 327–334. 18210139 10.1007/s00401-007-0337-z

[32] Nakamura M, Kaneko S, Ito H, Jiang S, Fujita K, et al (2013) Activation of transforming growth factor-beta/Smad signaling reduces aggregate formation of mislocalized TAR DNA-binding protein-43. Neurodegener Dis 11: 182–193. 22797246 10.1159/000338151

[33] Nakamura M, Kaneko S, Wate R, Asayama S, Nakamura Y, et al (2012) Regionally different immunoreactivity for Smurf2 and pSmad2/3 in TDP-43-positive inclusions of amyotrophic lateral sclerosis. Neuropathol Appl Neurobiol 10.1111/j.1365-2990.2012.01270.x 22435645

[34] Asai-Coakwell M, March L, Dai XH, Duval M, Lopez I, et al (2013) Contribution of growth differentiation factor 6-dependent cell survival to early-onset retinal dystrophies. Hum Mol Genet 22: 1432–1442. 23307924 10.1093/hmg/dds560

[35] Tierney KB (2011) Swimming performance assessment in fishes. J Vis Exp 10.3791/2572 PMC3197426 21633333

[36] Plaut I (2000) Effects of fin size on swimming performance, swimming behaviour and routine activity of zebrafish Danio rerio. J Exp Biol 203: 813–820. 10648223 10.1242/jeb.203.4.813

[37] McClelland GB, Craig PM, Dhekney K, Dipardo S (2006) Temperature- and exercise-induced gene expression and metabolic enzyme changes in skeletal muscle of adult zebrafish (Danio rerio). J Physiol 577: 739–751. 16990399 10.1113/jphysiol.2006.119032 PMC1890438

[38] Gilbert MJ, Zerulla TC, Tierney KB (2014) Zebrafish (Danio rerio) as a model for the study of aging and exercise: Physical ability and trainability decreases with age. Exp Gerontol 50C: 106–113 doi:10.1016/j.exger.2013.11.013 10.1016/j.exger.2013.11.013 24316042

[39] Asai-Coakwell M, French CR, Ye M, Garcha K, Bigot K, et al (2009) Incomplete penetrance and phenotypic variability characterize Gdf6-attributable oculo-skeletal phenotypes. Hum Mol Genet 18: 1110–1121. 19129173 10.1093/hmg/ddp008 PMC12118964

[40] Asai-Coakwell M, French CR, Berry KM, Ye M, Koss R, et al (2007) GDF6, a novel locus for a spectrum of ocular developmental anomalies. Am J Hum Genet 80: 306–315. 17236135 10.1086/511280 PMC1785352

[41] Tassabehji M, Fang ZM, Hilton EN, McGaughran J, Zhao Z, et al (2008) Mutations in GDF6 are associated with vertebral segmentation defects in Klippel-Feil syndrome. Hum Mutat 29: 1017–1027. 18425797 10.1002/humu.20741

[42] Settle SH Jr, Rountree RB, Sinha A, Thacker A, Higgins K, et al (2003) Multiple joint and skeletal patterning defects caused by single and double mutations in the mouse Gdf6 and Gdf5 genes. Dev Biol 254: 116–130. 12606286 10.1016/s0012-1606(02)00022-2

[43] Ye M, Berry-Wynne KM, Asai-Coakwell M, Sundaresan P, Footz T, et al (2010) Mutation of the bone morphogenetic protein GDF3 causes ocular and skeletal anomalies. Hum Mol Genet 19: 287–298. 19864492 10.1093/hmg/ddp496

[44] Farrell AP (2007) Comparisons of swimming performance in rainbow trout using constant acceleration and critical swimming speed tests. Journal of Fish Biology 72: 693–710.

[45] Tierney KB (2011) Behavioural assessments of neurotoxic effects and neurodegeneration in zebrafish. Biochim Biophys Acta 1812: 381–389. 21035547 10.1016/j.bbadis.2010.10.011

[46] Aberle H, Haghighi AP, Fetter RD, McCabe BD, Magalhaes TR, et al (2002) wishful thinking encodes a BMP type II receptor that regulates synaptic growth in Drosophila. Neuron 33: 545–558. 11856529 10.1016/s0896-6273(02)00589-5

[47] James RE, Broihier HT (2011) Crimpy inhibits the BMP homolog Gbb in motoneurons to enable proper growth control at the Drosophila neuromuscular junction. Development 138: 3273–3286. 21750037 10.1242/dev.066142 PMC3133917

[48] Chou HJ, Lai DM, Huang CW, McLennan IS, Wang HD, et al (2013) BMP4 is a peripherally-derived factor for motor neurons and attenuates glutamate-induced excitotoxicity in vitro. Plos One 8: e58441. 23472198 10.1371/journal.pone.0058441 PMC3589418

[49] Xiao L, Michalski N, Kronander E, Gjoni E, Genoud C, et al (2013) BMP signaling specifies the development of a large and fast CNS synapse. Nat Neurosci 16: 856–864. 23708139 10.1038/nn.3414

[50] Henriquez JP, Krull CE, Osses N (2011) The Wnt and BMP families of signaling morphogens at the vertebrate neuromuscular junction. Int J Mol Sci 12: 8924–8946. 22272112 10.3390/ijms12128924 PMC3257109

[51] Strelau J, Strzelczyk A, Rusu P, Bendner G, Wiese S, et al (2009) Progressive postnatal motoneuron loss in mice lacking GDF-15. J Neurosci 29: 13640–13648. 19864576 10.1523/JNEUROSCI.1133-09.2009 PMC3320210

[52] Katsuno M, Adachi H, Banno H, Suzuki K, Tanaka F, et al (2011) Transforming growth factor-beta signaling in motor neuron diseases. Curr Mol Med 11: 48–56. 21189118 10.2174/156652411794474356

[53] Zhang L, Lim SL, Du H, Zhang M, Kozak I, et al (2012) High temperature requirement factor A1 (HTRA1) gene regulates angiogenesis through transforming growth factor-beta family member growth differentiation factor 6. J Biol Chem 287: 1520–1526. 22049084 10.1074/jbc.M111.275990 PMC3256864

[54] Vawter MP, Dillon-Carter O, Tourtellotte WW, Carvey P, Freed WJ (1996) TGFbeta1 and TGFbeta2 concentrations are elevated in Parkinson's disease in ventricular cerebrospinal fluid. Exp Neurol 142: 313–322. 8934562 10.1006/exnr.1996.0200

[55] Li C, Zhao R, Gao K, Wei Z, Yin MY, et al (2011) Astrocytes: implications for neuroinflammatory pathogenesis of Alzheimer's disease. Curr Alzheimer Res 8: 67–80. 21143158 10.2174/156720511794604543

[56] Deininger M, Meyermann R, Schluesener H (1995) Detection of two transforming growth factor-beta-related morphogens, bone morphogenetic proteins-4 and -5, in RNA of multiple sclerosis and Creutzfeldt-Jakob disease lesions. Acta Neuropathol 90: 76–79. 7572083 10.1007/BF00294462

[57] Wyss-Coray T, Lin C, von Euw D, Masliah E, Mucke L, et al (2000) Alzheimer's disease-like cerebrovascular pathology in transforming growth factor-beta 1 transgenic mice and functional metabolic correlates. Ann N Y Acad Sci 903: 317–323. 10818521 10.1111/j.1749-6632.2000.tb06382.x

[58] Hashimoto Y, Chiba T, Yamada M, Nawa M, Kanekura K, et al (2005) Transforming growth factor beta2 is a neuronal death-inducing ligand for amyloid-beta precursor protein. Mol Cell Biol 25: 9304–9317. 16227582 10.1128/MCB.25.21.9304-9317.2005 PMC1265827

[59] Feng Z, Ko CP (2008) Schwann cells promote synaptogenesis at the neuromuscular junction via transforming growth factor-beta1. J Neurosci 28: 9599–9609. 18815246 10.1523/JNEUROSCI.2589-08.2008 PMC3844879

[60] Galbiati M, Onesto E, Zito A, Crippa V, Rusmini P, et al (2012) The anabolic/androgenic steroid nandrolone exacerbates gene expression modifications induced by mutant SOD1 in muscles of mice models of amyotrophic lateral sclerosis. Pharmacol Res 65: 221–230. 22178654 10.1016/j.phrs.2011.12.001 PMC3272141

[61] Perides G, Jensen FE, Edgecomb P, Rueger DC, Charness ME (1995) Neuroprotective effect of human osteogenic protein-1 in a rat model of cerebral hypoxia/ischemia. Neurosci Lett 187: 21–24. 7617293 10.1016/0304-3940(95)11327-s

[62] Hurley FM, Costello DJ, Sullivan AM (2004) Neuroprotective effects of delayed administration of growth/differentiation factor-5 in the partial lesion model of Parkinson's disease. Exp Neurol 185: 281–289. 14736509 10.1016/j.expneurol.2003.10.003

[63] Krieglstein K, Strelau J, Schober A, Sullivan A, Unsicker K (2002) TGF-beta and the regulation of neuron survival and death. J Physiol Paris 96: 25–30. 11755780 10.1016/s0928-4257(01)00077-8

[64] Tesla R, Wolf HP, Xu P, Drawbridge J, Estill SJ, et al (2012) Neuroprotective efficacy of aminopropyl carbazoles in a mouse model of amyotrophic lateral sclerosis. Proc Natl Acad Sci U S A 109: 17016–17021. 23027932 10.1073/pnas.1213960109 PMC3479516

[65] De Jesus-Cortes H, Xu P, Drawbridge J, Estill SJ, Huntington P, et al (2012) Neuroprotective efficacy of aminopropyl carbazoles in a mouse model of Parkinson disease. Proc Natl Acad Sci U S A 109: 17010–17015. 23027934 10.1073/pnas.1213956109 PMC3479520

[66] Ferraiuolo L, Kirby J, Grierson AJ, Sendtner M, Shaw PJ (2011) Molecular pathways of motor neuron injury in amyotrophic lateral sclerosis. Nat Rev Neurol 7: 616–630. 22051914 10.1038/nrneurol.2011.152

[67] Banyai L, Sonderegger P, Patthy L (2010) Agrin binds BMP2, BMP4 and TGFbeta1. Plos One 5: e10758. 20505824 10.1371/journal.pone.0010758 PMC2874008

[68] Westerfield M (2000) The zebrafish book. A guide for the laboratory use of zebrafish (Danio rerio). Eugene, OR: University of Oregon Press.

[69] Kaiser DM, Acharya M, Leighton PL, Wang H, Daude N, et al (2012) Amyloid beta precursor protein and prion protein have a conserved interaction affecting cell adhesion and CNS development. Plos One 7: e51305. 23236467 10.1371/journal.pone.0051305 PMC3517466

[70] Brett JR (1964) The respiratory metabolism and swimming performance of young sockeye salmon. Journal of the Fisheries Research Board of Canada 21: 1183–1226.

[71] Fraser B, Duval MG, Wang H, Allison WT (2013) Regeneration of cone photoreceptors when cell ablation is primarily restricted to a particular cone subtype. Plos One 8: e55410. 23383182 10.1371/journal.pone.0055410 PMC3559598

[72] Taylor WR, van Dyke GC (1985) Revised procedures for staining and clearing small fishes and other vertebrates for bone and cartilage study. Cybium 9: 107–119.

[73] Allison WT, Haimberger TJ, Hawryshyn CW, Temple SE (2004) Visual pigment composition in zebrafish: Evidence for a rhodopsin-porphyropsin interchange system. Vis Neurosci 21: 945–952. 15733349 10.1017/S0952523804216145

