# Supplementary figures and images for: Growth Differentiation Factor 6 As a Putative Risk Factor in Neuromuscular Degeneration
Source: PLoS One. 2014 Feb 28;9(2):e89183. doi: 10.1371/journal.pone.0089183 (PMC3938462; doi:10.1371/journal.pone.0089183)

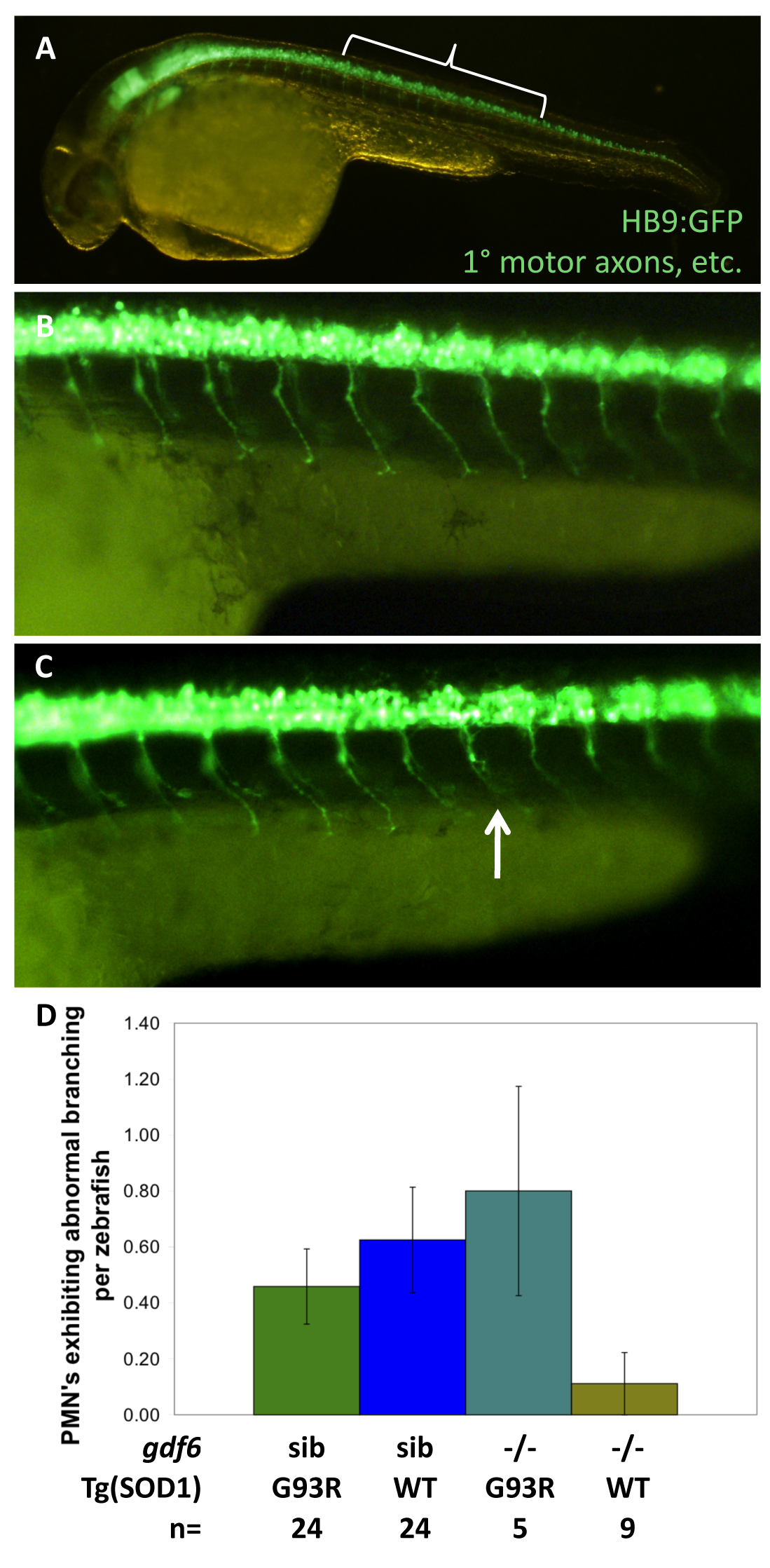

Supplement: Figure S1 — In larval zebrafish, mutations in gdf6a do not appreciably sensitize SOD1∧G93R zebrafish to develop ALS-like symptoms. Four genotypes combining Gdf6−/− alleles and SOD1∧G93R alleles were examined in Tg(HB9:eGFP) zebrafish expressing GFP in the axons of primary motor neurons (PMN), or via immunohistochemistry. A. Bracket indicates position of axons quantified, magnified in B,C. B. Normal primary motor axons. C. An example of an abnormal PMN axon (arrow). D. Quantification of primary motor axons in 30 hour post-fertilization embryosshow no difference based on gdf6a genotype; Thus effects are not developmental, and accord with a late-onset phenotype. Results indicated no significant effect of Gdf6 on the presence of axonopathies (p≥0.315, n≥5 larvae per genotype), although the highest rate of PMN axon abnormalities were observed in gdf6a−/−;SOD1+/G93R larvae. (TIF) [file pone.0089183.s001.tif]

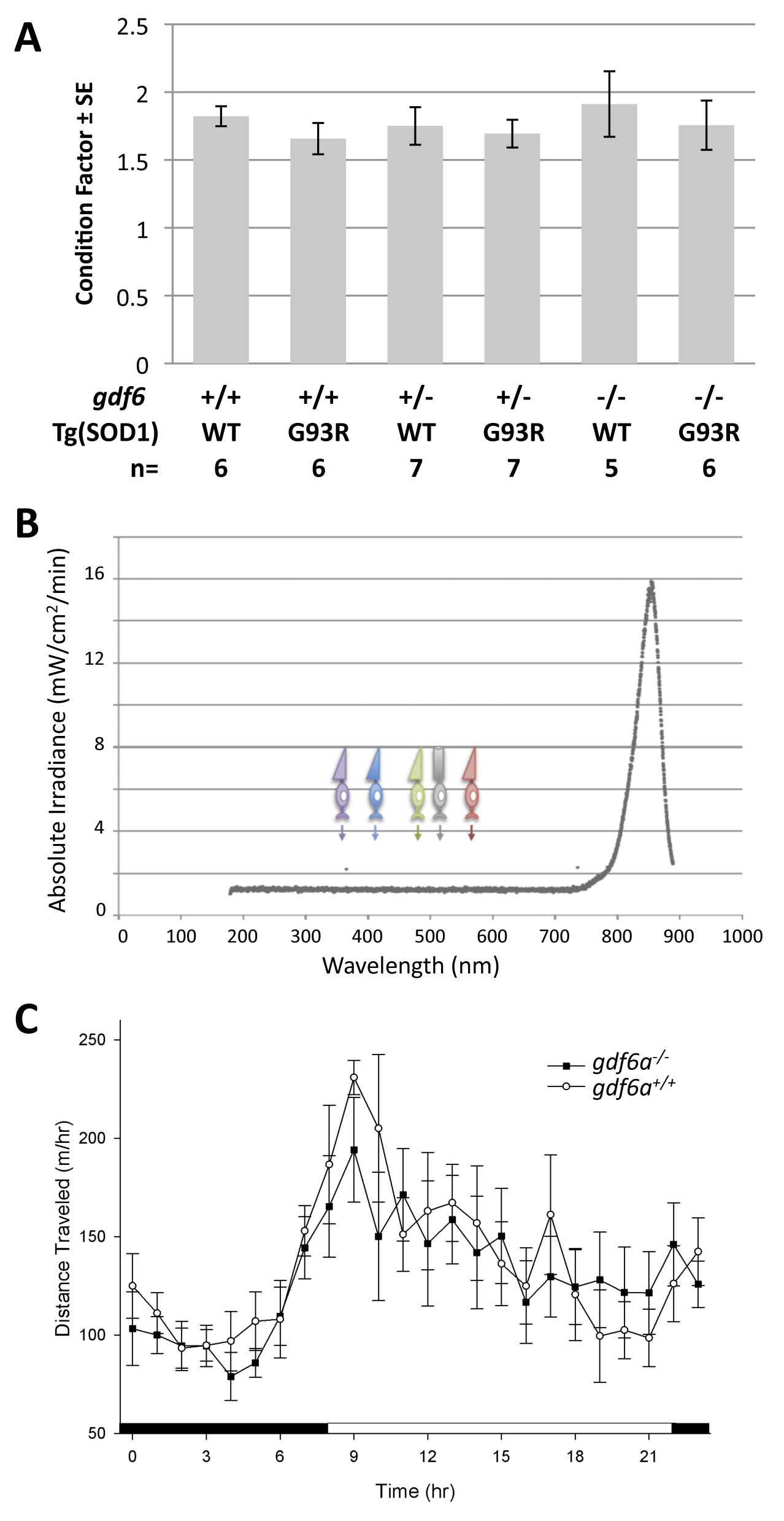

Supplement: Figure S2 — Eliminating alternate hypotheses that might account for differences in swimming behaviour between gdf6a−/− zebrafish and their siblings. Our overall conclusion is that gdf6a mutants have deficits in endurance due to a progressive loss of spinal motor neurons and disrupted neuromuscular junctions. Alternative explanations for these fish having reduced endurance are eliminated here. A. Body morphology was not significantly different based on genotype. Condition factor was determined using the standard formula from mass (g) and body length (BL, in mm), calculated as 100000Xmass/BL3. Condition factor did not vary based on genotype. Sample sizes (number of fish) indicated at the bottom of graph. B. Acute deficits in vision cannot account for differences between microphthalmic gdf6a−/− fish and their wildtype siblings. Infrared lighting conditions during behavioural tracking of zebrafish excludes a role for visual dysfunction in the assays of fish activity, power or endurance. Grey trace indicates photons available to fish during recording sessions. Arrows annotate the maximal wavelength of sensitivity of photoreceptors in zebrafish [73]: rod photoreceptors (grey) and cone photoreceptors (coloured to indicate spectral sensitivity, magenta for ultraviolet-sensitive cone, and blue-, green- and red-sensitive cones are indicated by the cognate colour) are documented. In sum, the infrared conditions used prevented vision from impacting behaviour of wild type or mutant fish the during tracking of fish movement in the open field test, endurance tests or sprint tests. C. gdf6a−/− zebrafish in open field test shows no significant difference in average movement compared to wild type siblings. Fish movement tracked over 24 hours, bar below abscissa indicates lights on and off. Mutant fish had near-normal activity levels throughout the circadian cycle. A lower average movement in mutants is noted immediately after the lights turn on (0800–0900 h, compared to siblings), though this di [file pone.0089183.s002.tif]
